# Algorithms for computing the double cut and join distance on both gene order and intergenic sizes

**DOI:** 10.1186/s13015-017-0107-y

**Published:** 2017-06-05

**Authors:** Guillaume Fertin, Géraldine Jean, Eric Tannier

**Affiliations:** 1 LS2N UMR CNRS 6004, Université de Nantes, 2 rue de la Houssinière, 44322 Nantes, France; 2 Institut National de Recherche en Informatique et en Automatique (Inria) Grenoble Rhône-Alpes, 655 avenue de l’Europe, 38330 Montbonnot-Saint-Martin, France; 3 CNRS, Laboratoire de Biomètrie et Biologie Evolutive UMR5558, Univ Lyon, Université Lyon 1, 43 boulevard du 11 novembre 1918, 69622 Villeurbanne, Villeurbanne France

**Keywords:** DCJ, Intergenic regions, Genome rearrangements, Algorithms

## Abstract

**Background:**

Combinatorial works on genome rearrangements have so far ignored the influence of intergene sizes, i.e. the number of nucleotides between consecutive genes, although it was recently shown decisive for the accuracy of inference methods (Biller et al. in Genome Biol Evol 8:1427–39, 2016; Biller et al. in Beckmann A, Bienvenu L, Jonoska N, editors. Proceedings of Pursuit of the Universal-12th conference on computability in Europe, CiE 2016, Lecture notes in computer science, vol 9709, Paris, France, June 27–July 1, 2016. Berlin: Springer, p. 35–44, 2016). In this line, we define a new genome rearrangement model called wDCJ, a generalization of the well-known double cut and join (or DCJ) operation that modifies both the gene order and the intergene size distribution of a genome.

**Results:**

We first provide a generic formula for the wDCJ distance between two genomes, and show that computing this distance is strongly NP-complete. We then propose an approximation algorithm of ratio 4/3, and two exact ones: a fixed-parameter tractable (FPT) algorithm and an integer linear programming (ILP) formulation.

**Conclusions:**

We provide theoretical and empirical bounds on the expected growth of the parameter at the center of our FPT and ILP algorithms, assuming a probabilistic model of evolution under wDCJ, which shows that both these algorithms should run reasonably fast in practice.

## Background

### General context

Mathematical models for genome evolution by rearrangements have defined a genome as a linear or circular ordering of genes^1^ [1]. These orderings have first been seen as (possibly signed) permutations, or strings if duplicate genes are present, or disjoint paths and cycles in graphs in order to allow multiple chromosomes. However, the organization of a genome is not entirely subsumed in gene orders. In particular, consecutive genes are separated by an intergenic region, and intergenic regions have diverse sizes [2]. Besides, it was recently shown that integrating intergene sizes in the models radically changes the distance estimations between genomes, as usual rearrangement distance estimators ignoring intergene sizes do not estimate well on realistic data [3, 4]. We thus propose to re-examine the standard models and algorithms in this light. A first step is to define and compute standard distances, such as double cut and join (or DCJ) [5], taking into account intergene sizes. In this setting, two genomes are considered, which are composed of gene orders *and* intergene sizes. One is transformed into the other by wDCJ operations, where additionally the sizes of the intergenes it affects can be modified.

### Genomes and rearrangements

Given a set *V* of vertices such that $|V|=2n$, we define a *genome* *g* as a set of *n* disjoint edges, i.e. a perfect matching on *V*. A genome is *weighted* if each edge *e* of *g* is assigned an integer weight $w(e)\ge 0$, and we define *W*(*g*) as the sum of all weights of the edges of *g*. The union of two genomes $g_1$ and $g_2$ on the same set *V* thus forms a set of disjoint even size cycles called the *breakpoint graph* $BG(g_1, g_2)$ of $g_1$ and $g_2$, in which each cycle is *alternating*, i.e. is composed of edges alternately belonging to $g_1$ and $g_2$. Note that in the rest of the paper, we will be only interested in evenly weighted genomes, *i.e.* genomes $g_1$ and $g_2$ such that $W(g_1)=W(g_2)$.

A *Double cut-and-join* (DCJ) [5] is an operation on an unweighted genome *g*, which transforms it into another genome $g'$ by deleting two edges *ab* and *cd* and by adding either (i) edges *ac* and *bd*, or (ii) edges *ad* and *bc*. If *g* is weighted, the operation we introduce in this paper is called wDCJ: wDCJ is a DCJ that additionally modifies the weights of the resulting genome in the following way: if we are in case (i), (1) any edge but *ac* and *bd* is assigned the same weight as in *g*, and (2) *w*(*ac*) and *w*(*bd*) are assigned arbitrary non negative integer weights, with the constraint that $w(ac)+w(bd)=w(ab)+w(cd)$. If we are in case (ii), a similar rule applies by replacing *ac* by *ad* and *bd* by *bc*. Note that wDCJ clearly generalizes the usual DCJ, since any unweighted genome *g* can be seen as a weighted one in which $w(e)=0$ for any edge *e* in *g*.

### Motivation for these definitions

This representation of a genome supposes that each vertex is a *gene extremity* (a gene being a segment, it has two extremities, which explains the even number of vertices), and an edge means that two gene extremities are contiguous on a chromosome. This representation generalizes signed permutations, and allows for an arbitrary number of circular and linear chromosomes. The fact that there should be *n* edges in a genome means that chromosomes are circular, or that extremities of linear chromosomes are not in the vertex set. It is possible to suppose so when the genomes we compare are *co-tailed*, i.e. the same gene extremities are extremities of chromosomes in both genomes. In this way, a wDCJ on a circular (resp. co-tailed) genome always yields a circular (resp. co-tailed) genome, which, in our terminology, just means that a weighted perfect matching stays a weighted perfect matching through wDCJ. So all along this paper we suppose that we are in the particular case of classical genomic studies where genomes are co-tailed or circular. Each edge represents an intergenic region. Weights on edges are intergene sizes, that is, the number of nucleotides separating two genes. The way weights are distributed after a wDCJ models a breakage inside an intergene between two nucleotides.

### Statement of the problem

Given two evenly weighted genomes $g_1$ and $g_2$ on the same set *V* of 2*n* vertices, a sequence of wDCJ that transforms $g_1$ into $g_2$ is called a *wDCJ sorting scenario*. Note that any sequence transforming $g_1$ into $g_2$ can be easily transformed into a sequence of same length transforming $g_2$ into $g_1$, as the problem is fully symmetric. Thus, in the following, we will always suppose that $g_2$ is fixed and that the wDCJ are applied on $g_1$. The wDCJ *distance* between $g_1$ and $g_2$, denoted $wDCJ(g_1, g_2)$, is defined as the number of wDCJ of a shortest wDCJ sorting scenario. Note that when genomes are unweighted, computing the usual DCJ distance is tractable, as $DCJ (g_1, g_2)=n-c$, where *c* is the number of cycles of $BG(g_1, g_2)$ [5]. The problem we consider in this paper, that we denote by wDCJ-dist, is the following: given two evenly weighted genomes $g_1$ and $g_2$ defined on the same set *V* of 2*n* vertices, determine $wDCJ(g_1, g_2)$.

We need further notations. The *imbalance* of a cycle *C* in $BG(g_1, g_2)$ is denoted *I*(*C*), and is defined as follows: $I(C)=w_1(C)-w_2(C)$, where $w_1(C)$ (resp. $w_2(C)$) is the sum of the weights of the edges of *C* which belong to $g_1$ (resp. $g_2$). A cycle *C* of the breakpoint graph is said to be *balanced* if $I(C)=0$, and *unbalanced* otherwise. We will denote by $\mathcal {C}_u$ the set of unbalanced cycles in $BG(g_1,g_2)$, and by $n_u=|\mathcal {C}_u|$ its cardinality. Similarly, $n_b$ denotes the number of balanced cycles in $BG(g_1, g_2)$, and $c=n_u+n_b$ denotes the (total) number of cycles in $BG(g_1,g_2)$.

A problem *P* is said to be fixed-parameter tractable (or FPT) with respect to a parameter *k* if it can be solved exactly in $O(f(k)\cdot poly(n))$ time, where *f* is any computable function, *n* is the size of the input, and *poly*(*n*) is a polynomial function of *n*. FPT algorithms are usually sought for NP-hard problems: if *P* is proved to be FPT in *k*, then the exponential part of the running time for solving *P* is confined to parameter *k*. Hence, if *k* is small in practice, *P* can still be solved exactly in reasonable time. Note also that the running time $O(f(k)\cdot poly(n))$ is often written $O^*(f(k))$, where the polynomial factor is omitted.

### Related works

Several generalizations or variants of standard genome rearrangement models integrate more realistic features in order to be closer to real genome evolution. It concerns, among others, models where inversions are considered, that are weighted by their length or symmetry around a replication origin [6], by the proximity of their extremities in the cell [7], or by their use of hot regions for rearrangement breakages [8]. Genome rearrangement taking into account intergenic sizes have been introduced in [3]. Their ability to capture realistic features has been demonstrated in [3, 4], while a variant of the wDCJ distance has been recently published [9]. The model in [9] is however different from ours, as it allows indels and uses a different distance definition. The present article is an extended version of [10] that includes full proofs, improves the approximation ratio for wDCJ-dist and considers several parameters for the FPT complexity.

### Our results

In this paper, we explore the algorithmic properties of wDCJ-dist. We first provide the main properties of (optimal) wDCJ sorting scenarios in “Main properties of sorting by wDCJ”. We then show in “Algorithmic aspects of wDCJ-dist'' that the wDCJ-dist problem is strongly NP-complete, 4/3 approximable, and we provide two exact algorithms, in the form of an FPT algorithm and an ILP (Integer Linear Programming) formulation. By simulations and analytic studies on a probabilistic model of genome evolution, in “A probabilistic model of evolution by wDCJ” we bound the parameter at the center of both our FPT and ILP algorithms, and conclude that they should run reasonably fast in practice.

## Main properties of sorting by wDCJ

The present section is devoted to providing properties of any (optimal) wDCJ sorting scenario. These properties mainly concern the way the breakpoint graph evolves, whenever one or several wDCJ is/are applied. These will lead to a closed-form expression for the wDCJ distance (Theorem 7). Moreover, they will also be essential in the algorithmic study of the wDCJ-dist problem that will be developed in “Main properties of sorting by wDCJ''. We first show the following lemma.

### **Lemma 1**

*Let C be a balanced cycle of some breakpoint graph* $BG(g_1,g_2)$ *. Then there exist three consecutive edges e,* *f*, *g in* *C such that (i)* *e and* *g belong to* $g_1$ *and (ii)* $w(e)+w(g)\ge w(f)$.

### *Proof*

Suppose, aiming at a contradiction, that for any three consecutive edges *e*, *f*, *g* in *C* with $e,g\in E(g_1)$, we have $w(e)+w(g)< w(f)$. Summing this inequality over all such triplets of consecutive edges of *C*, we obtain the following inequality: $2\cdot w_1(C) < w_2(C)$. Since *C* is balanced, by definition we have $w_1(C)-w_2(C)=0$. Hence we obtain $w_1(C)<0$, a contradiction since all edge weights are non negative by definition. $\square$

Note that any wDCJ can act on the number of cycles of the breakpoint graph in only three possible ways: either this number is increased by one (cycle *split*), decreased by one (cycle *merge*), or remains the same (cycle *freeze*). We now show that if a breakpoint graph only contains balanced cycles, then any optimal wDCJ sorting scenario only uses cycle splits.

### **Proposition 2**

*Let* $BG(g_1, g_2)$ *be a breakpoint graph that contains balanced cycles only – in which case* $c=n_b$.* Then* $wDCJ(g_1, g_2)=n-n_b$.

### *Proof*

First note that for any two genomes $g_1$ and $g_2$, we have $wDCJ(g_1,g_2)\ge n-c$, because the number of cycles can increase by at most one after each wDCJ. In our case, $c=n_b$, thus it suffices to show here that $wDCJ(g_1,g_2)\le n-n_b$ to conclude. We will show that whenever $g_1\ne g_2$, there always exists a wDCJ transforming $g_1$ into $g'_1$ such that (i) $BG(g'_1,g_2)$ only contains balanced cycles and (ii) $n'_b=n_b +1$, where $n'_b$ is the number of cycles in $BG(g'_1,g_2)$. For this, assume $g_1\ne g_2$ ; then there exists a balanced cycle *C* of (even) length $m\ge 4$ in $BG(g_1,g_2)$. By Lemma 1, we know there exist in *C* three consecutive edges *e*, *f*, *g* such that $w(e)+w(g)\ge w(f)$. Let $e=ab$, $f=bc$ and $g=cd$. The wDCJ we apply is the following: cut *ab* and *cd*, then join *ad* and *bc*. This transforms *C* into a new cycle $C'$ whose length is $m-2$, and creates a new 2-cycle $C''$ whose endpoints are *b* and *c*. The newly created edge *bc* is assigned a weight equal to *w*(*f*), which is possible since by Lemma 1, $w(ab)+w(cd) \ge w(f)$. Moreover, by definition of a wDCJ, the weight of the newly created edge *ad* satisfies $w(ad)=w(e)+w(g)-w(f)$. Thus, by Lemma 1, $w(ad)\ge 0$. Finally, because *C* and $C''$ are balanced, and because $w_1(C)=w_1(C')+w_1(C'')$ [resp. $w_2(C)=w_2(C')+w_2(C'')$], necessarily $C'$ is balanced too.

Thus, since such a wDCJ keeps all the cycles balanced while increasing the number of cycles by one, we can apply it iteratively until we reach the point where all cycles are of length 2, i.e. the two genomes are equal. This shows that $wDCJ(g_1,g_2)\le n-n_b$, and the result is proved. $\square$

In the following, we are interested in the sequences of two wDCJ formed by a cycle split *s* *directly followed by a cycle merge* *m*, to the exception of *df-sequences* (for *double-freeze*), which is the special case where *s* is applied on a cycle *C* (forming cycles $C_a$ and $C_b$) and *m* merges back $C_a$ and $C_b$ to give a new cycle $C'$ built on the same set of vertices as *C*. The name derives from the fact that a *df-sequence* acts as a freeze, except that it can involve up to four edges in the cycle, as opposed to only two edges for a freeze.

### **Proposition 3**

*In a wDCJ sorting scenario, if there is a sequence of two operations formed by a cycle split s directly followed by a cycle merge m that is not a df-sequence, then there exists a wDCJ sorting scenario of same length where s and m are replaced by a cycle merge* $m'$ *followed by a cycle split* $s'$.

### *Proof*

Let *s* and *m* be two consecutive wDCJ in a sorting scenario that do not form a df-sequence, where *s* is a split, *m* is a merge, and *s* is applied before *m*. Let also *G* (resp. $G'$) be the breakpoint graph before *s* (resp. after *m*) is applied. We will show that there always exist two wDCJ $m'$ and $s'$, such that (i) $m'$ is a cycle merge, (ii) $s'$ is a cycle split and (iii) starting from *G*, applying $m'$ then $s'$ gives $G'$. First, if none of the two cycles produced by *s* is used by *m*, then the two wDCJ are independent, and it suffices to set $m'=m$ and $s'=s$ to conclude.

**Fig. 1:**
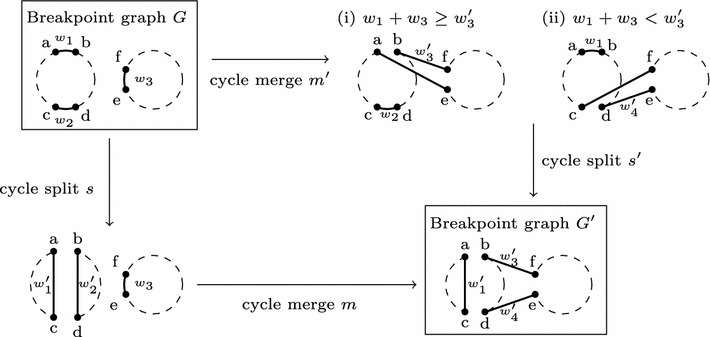
Two different scenarios that lead to $G'$ starting from *G*: (*downward*) a split *s* followed by a merge *m* ; (*rightward*) a merge $m'$ followed by a split $s'$

Now suppose one of the two cycles produced by *s* is involved in *m*. Let $C_1$ denote the cycle on which *s* is applied, and let us assume *s* cuts *ab* and *cd*, of respective weights $w_1$ and $w_2$, and joins *ac* and *bd*, of respective weights $w'_1$ and $w'_2$ — thus $w_1 + w_2 = w'_1 + w'_2$ (a). We will denote by $C_a$ (resp. $C_b$) the two cycles obtained by *s* from $C_1$ ; see Fig.  1 for an illustration. Now let us consider *m*. Wlog, let us suppose that *m* acts on $C_b$ and another cycle $C_2 \ne C_a$ (since df-sequences are excluded), in order to produce cycle $C_3$. It is easy to see that if *m* cuts an edge different from *bd* in $C_b$, then *s* and *m* are two independent wDCJ, and thus can be safely swapped. Thus we now assume that *m* cuts *bd*. Suppose the edge that is cut in $C_2$ is *ef*, of weight $w_3$, and that the joins are edges *bf* and *de*, of respective weights $w'_3$ and $w'_4$. We thus have $w'_3 + w'_4 = w'_2 + w_3$ (b). Moreover, adding (a) and (b) gives $w_1 + w_2 + w_3 = w'_1 + w'_3 + w'_4$ (c). Now let us show that there exists a scenario that allows to obtain $C_a$ and $C_3$ from $C_1$ and $C_2$, which begins by a merge followed by a split. For this, we consider two cases:

- $w_1 + w_3 \ge w'_3$ [see Fig.  1(i)]: $m'$ consists in cutting *ab* from $C_1$ and *ef* from $C_2$, then forming *ae* and *bf*, so as to obtain a unique cycle *C*. Note that *C* now contains edges *cd* (of weight $w_2$), *bf* (of weight $w'_3$) and *ae* (of weight $w_1+w_3-w'_3$, which is non negative by hypothesis). Then, $s'$ is defined as follows: cut *ae* and *cd*, form edges *ac*, *de*. Finally, note that assigning $w'_1$ to *ac* and $w'_4$ to *de* is possible, since *ae* is of weight $w_1+w_3-w'_3$, *cd* is of weight $w_2$, and since $w_1+w_3-w'_3+w_2=w'_1 + w'_4$ by (c).
- $w_1 + w_3 < w'_3$ [see Fig. 1(ii)]. Consider the following merge $m'$: cut edges *cd* and *ef*, and form the edges *de* of weight $w'_4$, and *cf* of weight $w=w_2+w_3-w'_4$. This merge is feasible because $w\ge 0$: indeed, by hypothesis $w_1 + w_3 < w'_3$, i.e. $w_1 + w_2 + w_3 < w_2 + w'_3$, which by (c) implies $w'_1+w'_4<w_2$. Thus $w'_4<w_2$, and consequently $w>w_3\ge 0$. Now let $s'$ be as follows: cut *ab* (of weight $w_1$) and *cf* (of weight $w=w_2+w_3-w'_4$) to form edges *ac* and *bf* of respective weights $w'_1$ and $w'_3$. Note that $s'$ is always feasible since $w_1+w = w_1 + w_2 + w_3 - w'_4 = w'_1 + w'_3$ by (c).

In all cases, it is always possible to obtain $G'$, starting from *G*, using a merge $m'$ followed by a split $s'$, rather than *s* followed by *m*, and the result is proved. $\square$

### **Proposition 4**

*In an optimal wDCJ sorting scenario, no cycle freeze or df-sequence occurs.*

### *Proof*

Suppose a wDCJ sorting scenario contains at least one cycle freeze or df-sequence, and let us consider the last such event *f* that appears in it. We will show that there also exists a sorting scenario that does not contain *f*, and whose length is decreased by at least one. For this, note that the sequence of wDCJ that follow *f*, say $\mathcal {S}$, is only composed of cycle splits and merges which do not form df-sequences. By Proposition 3, in $\mathcal {S}$ any split that precedes a merge can be replaced by a merge that precedes a split, in such a way that the new scenario is a sorting one, and of same length. By iterating this process, we end up with a sequence $\mathcal {S'}$ in which, after *f*, we operate a series *M* of merges, followed by a series *S* of splits. Let $G_M$ be the breakpoint graph obtained after all *M* merges are applied. If a cycle was unbalanced in $G_M$, any split would leave at least one unbalanced cycle, and it would be impossible to finish the sorting by applying the splits in *S*. Thus $G_M$ must contain only balanced cycles. Recall that *f* acts inside a given cycle *C*, while maintaining its imbalance *I*(*C*) unchanged. *C* may be iteratively merged with other cycles during *M*, but we know that, in $G_M$, the cycle $C'$ that finally “contains” *C* is balanced. Thus, if we remove *f* from the scenario, the breakpoint graph $G'_M$ we obtain only differs from $G_M$ by the fact that $C'$ is now replaced by another cycle $C''$, which contains the same vertices and is balanced. However, by Proposition 2, we know that $G'_M$ can be optimally sorted using the same number of splits as $G_M$, which allows us to conclude that there exists a shorter sorting scenario that does not use *f*. $\square$

### **Proposition 5**

*Any wDCJ sorting scenario can be transformed into another wDCJ sorting scenario of same or shorter length, and in which any cycle merge occurs before any cycle split.*

### *Proof*

By Proposition 4, we can transform any sorting scenario into one of same or shorter length that contains no cycle freeze nor df-sequence. Moreover, by Proposition 3, if there exist two consecutive wDCJ which are respectively a cycle split and a cycle merge, they can be replaced by a cycle merge followed by a cycle split, leading to a scenario that remains sorting and of same length. Thus, it is possible to iterate such an operation until no cycle split is directly followed by a cycle merge, *i.e.* all merges are performed before all splits. $\square$

### **Proposition 6**

*In an optimal wDCJ sorting scenario, no balanced cycle is ever merged.*

### *Proof*

We know that no optimal wDCJ scenario contains a cycle freeze or a df-sequence (Proposition 4). We also can assume that the scenario is such that all merges appear before all splits (Proposition 5). Let *M* (resp. *S*) be the sequence of merges (resp. splits) in this scenario. Let us suppose that at least one balanced cycle is merged in this scenario, and let us observe the last such merge *m*. Among the two cycles that are merged during *m*, at least one, say $C_1$, is balanced. Let us call $C'_1$ the cycle that “contains” $C_1$ after *M* is applied, and let $G_M$ be the breakpoint graph obtained after *M* is applied. We know that $G_M$ only contains balanced cycles, as no split can generate two balanced cycles from an unbalanced one. In particular, $C'_1$ is balanced. Let *c* denote the number of cycles in $G_M$. We know by Proposition 2 that it takes exactly $n-c$ wDCJ to sort $G_M$, leading to a scenario of length $l=|M|+n-c$. Now, if we remove *m* from *M* and look at the graph $G'_M$ obtained after all merges are applied, $G'_M$ contains the same cycles as $G_M$, except that $C'_1$ is now “replaced” by two balanced cycles $C''_1$ and $C_1$, where the vertices of $C'_1$ are the same as the ones from $C''_1$ and $C_1$. Thus, by Proposition 2, it takes exactly $n-(c+1)$ wDCJ to sort $G'_M$, which leads to a scenario of length $l'=|M|-1+n-(c+1)=l-2$ and contradicts the optimality of the initial scenario. Hence *m* does not happen in an optimal wDCJ sorting scenario, and the proposition is proved. $\square$

Based on the above results, we are now able to derive a formula for the wDCJ distance, which is somewhat similar to the “classical” DCJ distance formula [5].

### **Theorem 7**

*Let* $BG(g_1,g_2)$ *be the breakpoint graph of two genomes* $g_1$ *and* $g_2$ *, and let c be the number of cycles in* $BG(g_1,g_2)$.* Then* $wDCJ(g_1,g_2)=n-c+2m$ *, where m is the minimum number of cycle merges needed to obtain a set of balanced cycles from the unbalanced cycles of* $BG(g_1,g_2)$.

### *Proof*

By the previous study, we know that there exists an optimal wDCJ scenario without cycle freezes or df-sequences, and in which merges occur before splits (Propositions 4,  5). We also know that before the splits start, the graph $G_M$ we obtain is a collection of balanced cycles, and that the split sequence that follows is optimal and only creates balanced cycles (Proposition 2). Thus the optimal distance is obtained when the merges are as few as possible. By Proposition 6, we know that no balanced cycle is ever used in a cycle merge in an optimal scenario. Hence an optimal sequence of merges consists in creating balanced cycles from the unbalanced cycles of $BG(g_1,g_2)$ only, using a minimum number *m* of merges. Altogether, we have (i) *m* merges that lead to $c-m$ cycles, then (ii) $n-(c-m)$ splits by Proposition 2. Hence the result. $\square$

## Algorithmic aspects of wDCJ-dist

Based on the properties of a(n optimal) wDCJ sorting scenario given in “Main properties of sorting by wDCJ'', we are now able to provide algorithmic results concerning the wDCJ-dist problem.

### Complexity of wDCJ-dist

The computational complexity of wDCJ-dist is given by the following theorem. As there are numerical values in the input of wDCJ-dist, the complexity has to be established in a weak or strong form, i.e. considering numbers in the input in binary or unary notation.

#### **Theorem 8**

*The wDCJ-* dist *problem is strongly* NP-*complete*.

#### *Proof*

The proof is by reduction from the strongly NP-complete 3-Partition problem [11], whose instance is a multiset $A=\{a_1,a_2\ldots a_{3n}\}$ of 3*n* positive integers such that (i) $\sum _{i=1}^{3n} a_i=B\cdot n$ and (ii) $\frac{B}{4}< a_i< \frac{B}{2}$ for any $1\le i \le 3n$, and where the question is whether one can partition *A* into *n* multisets $A_1\ldots A_n$, such that for each $1\le i\le n$, $\sum _{a_j\in A_i} a_j=B$. Given any instance *A* of 3-Partition, we construct two genomes $g_1$ and $g_2$ as follows: $g_1$ and $g_2$ are built on a vertex set *V* of cardinality 8*n*, and consist of the same perfect matching. Thus $BG(g_1, g_2)$ is composed of 4*n* trivial cycles, that is cycles of length 2, say $C_1,C_2\ldots C_{4n}$. The only difference between $g_1$ and $g_2$ thus lies on the weights of their edges. For any $1\le i\le 4n$, let $e_i^1$ (resp. $e_i^2$) be the edge from $C_i$ that belongs to $g_1$ (resp. $g_2$). The weight we give to each edge is the following: for any $1\le i\le 3n$, $w(e_i^1)=a_i$ and $w(e_i^2)=0$; for any $3n+1\le i\le 4n$, $w(e_i^1)=0$ and $w(e_i^2)=B$. As a consequence, the imbalance of each cycle is $I(C_i)=a_i$ for any $1\le i\le 3n$, and $I(C_i)=-B$ for any $3n+1\le i\le 4n$. Now we will prove the following equivalence: 3-Partition is satisfied iff $wDCJ(g_1, g_2)\le 6n$.

$(\Rightarrow )$ Suppose there exists a partition $A_1\ldots A_n$ of *A* such that for each $1\le i\le n$, $\sum _{a_j\in A_i} a_j=B$. For any $1\le i\le n$, let $A_i=\{a_{i_1},a_{i_2},a_{i_3}\}$. Then, for any $1\le i\le n$, we merge cycles $C_{i_1}$, $C_{i_2}$ and $C_{i_3}$, then apply a third merge with $C_{3n+i}$. For each $1\le i\le n$, these three merges lead to a balanced cycle, since after the two first merges, the obtained weight is $a_{i_1}+a_{i_2}+a_{i_3}=B$. After these 3*n* merges (in total) have been applied, we obtain *n* balanced cycles, from which $4n-n=3n$ splits suffice to end the sorting, as stated by Proposition 2. Thus, altogether we have used 6*n* wDCJ, and consequently $wDCJ(g_1,g_2)\le 6n$.

$(\Leftarrow )$ Suppose that $wDCJ(g_1, g_2)\le 6n$. Recall that in the breakpoint graph $BG(g_1,g_2)$, we have $c=4n$ cycles and 8*n* vertices. Thus, by Theorem 7, we know that $wDCJ(g_1, g_2)=4n-4n+2m=2m$, where *m* is the smallest number of merges that are necessary to obtain a set of balanced cycles from $BG(g_1, g_2)$. Since we suppose $wDCJ(g_1, g_2)\le 6n$, we conclude that $m\le 3n$. Otherwise stated, the number of balanced cycles we obtain after the merges cannot be less than *n*, because we start with 4*n* cycles and apply at most 3*n* merges. However, at least four cycles from $C_1,C_2\ldots C_{4n}$ must be merged in order to obtain a single balanced cycle: at least three from $C_1,C_2\ldots C_{3n}$ (since any $a_i$ satisfies $\frac{B}{4}< a_i< \frac{B}{2}$ by definition), and at least one from $C_{3n+1},C_{3n+2}\ldots C_{4n}$ (in order to end up with an imbalance equal to zero). Thus any balanced cycle is obtained using exactly four cycles (and thus three merges), which in turn implies that there exists a way to partition the multiset *A* into $A_1\ldots A_n$ in such a way that for any $1\le i\le n$, $(\sum _{a_j\in A_i})-B=0$, which positively answers the 3-Partition problem. $\square$

### Approximating wDCJ-dist

Since wDCJ-dist is NP-complete, we now look for algorithms that approximately compute the wDCJ distance. We first begin by the following discussion: let $g_1$ and $g_2$ be two evenly weighted genomes, where $\mathcal {C}_u=\{C_1,C_2\ldots C_{n_u}\}$ is the set of unbalanced cycles in $BG(g_1, g_2)$. It can be seen that any optimal solution for wDCJ-dist will be obtained by merging a maximum number of pairs of cycles $\{C_i,C_j\}$ from $\mathcal {C}_u$ such that $I(C_i)+I(C_j)=0$, because each such pair represents two unbalanced cycles that become balanced when merged. Let $S_2=\{C_{i_1},C_{i_2}\ldots C_{i_{n_2}}\}$ be a maximum cardinality subset of $\mathcal {C}_u$ such that $I(C_{i_j})+I(C_{i_{j+1}})=0$ for any odd *j*, $1\le j< n_2$: $S_2$ thus contains a maximum number of cycles that become balanced when merged by pairs. Note that $S_2$ can be easily computed by a greedy algorithm that iteratively searches for a number and its opposite among the imbalances in $\mathcal {C}_u$. Now, $\mathcal {C'}_u=\mathcal {C}_u \setminus S_2$ needs to be considered. It would be tempting to go one step further by trying to extract from $\mathcal {C'}_u$ a maximum number of *triplets* of cycles whose imbalances sum to zero. This leads us to define the following problem:

#### Max-Zero-Sum-Triplets (MZS3)

**Instance**: A multiset $\mathcal {P}=\{p_1,p_2\ldots p_n\}$ of numbers $p_i\in \mathbb {Z}^*$ such that for any $1\le i,j\le n$, $p_i+p_j\ne 0$.

**Output**: A maximum cardinality set $\mathcal {P'}$ of non intersecting triplets from $\mathcal {P}$, such that each sums to zero.

Note that the multiset $\mathcal {P}$ in the definition of MZS3 corresponds to the multiset of imbalances of $\mathcal {C'}_u$ in wDCJ-dist. The next two propositions (Propositions 9,  10) consider resp. the computational complexity and approximability of MZS3. The latter will be helpful for devising an approximation algorithm for wDCJ-dist, as shown in Theorem 11 below.

##### **Proposition 9**

*The* MZS3* problem is strongly* NP *-complete*.

##### *Proof*

The proof is by reduction from Numerical 3-Dimensional Matching (or N3DM), a decision problem defined as follows: given three multisets of positive integers *W*, *X* and *Y* containing *m* elements each, and a positive integer *b*, does there exist a set of triplets $T\subseteq W \times X \times Y$ in which every integer from *W*, *X*, *Y* appears in exactly one triplet from *T*, and such that for every triplet $\{w,x,y\}\in T$, $w+x+y=b$? The N3DM problem has been proved to be strongly NP-complete in [11]. Note that, in addition, we can always assume that any element *s* in *W*, *X* or *Y* satisfies $s<b$, otherwise the answer to N3DM is clearly negative.

Given a set *S* of integers and an integer *p*, we denote by $S+p$ (resp. $S-p$) the set containing all elements of *S* to which *p* has been added (resp. subtracted). Given any instance $I=\{W,X,Y,b\}$ of N3DM, we construct the following instance of MZS3: $I'=\mathcal {P}=(W+b)\cup (X+3b)\cup (Y-5b)$. Note that $\mathcal {P}$ contains $n=3m$ elements that all strictly lie between $-5b$ and 4*b* ; thus the input size of $I'$ does not exceed a constant times the input size of *I*. Note also that no two elements $s,t\in \mathcal {P}$ are such that $s+t=0$, because each negative (resp. positive) element in $\mathcal {P}$ is strictly less than $-4b$ (resp. than 4*b*).

We now claim that the answer to N3DM on *I* is positive iff MZS3 outputs exactly $m=\frac{n}{3}$ independent triplets, each summing to zero.

($\Rightarrow$) Suppose the answer to N3DM on *I* is positive, and let *T* be the output set. The answer to MZS3 is constructed as follows: for any triplet $\{w,x,y\}$ that sums to zero in *T*, add $\{w+b,x+3b,y-5b\}$ to $\mathcal {P'}$. Since *T* covers all elements from *W*, *X* and *Y* exactly once, then $\mathcal {P'}$ contains exactly $m=\frac{n}{3}$ non intersecting triplets. Besides, each triplet sums to $(w+b)+(x+3b)+(y-5b)=(x+y+w)-b=0$ since $x+y+w=b$ by assumption.

($\Leftarrow$) Suppose there exist $\frac{n}{3}$ non intersecting triplets $\{f_i,g_i,h_i\}$ in $\mathcal {P}$, $1\le i\le \frac{n}{3}$ such that $f_i+g_i+h_i=0$. Our goal is to show that (wlog) $f_i\in W+b, g_i\in X+3b$ and $h_i\in Y-5b$. As mentioned above, we can assume that any element in *W*, *X*, *Y* strictly lies between 0 and *b*. Thus we have the following set of inequalities:

- any element $w\in (W+b)$ satisfies $b<w<2b$
- any element $x\in (X+3b)$ satisfies $3b<x<4b$
- any element $y\in (Y-5b)$ satisfies $-5b<y<-4b$

It can be seen from the above inequalities that any triplet that sums to zero must take one value in each of the sets $(W+b)$, $(X+3b)$ and $(Y-5b)$ (otherwise the sum is either strictly negative or strictly positive). Thus, for each $\{f_i,g_i,h_i\}$ returned by MZS3, we add $\{f'_i,g'_i,h'_i\}=\{(f_i-b),(g_i-3b),(h_i+5b)\}$ to *T*. We now claim that *T* is a positive solution to N3DM: each triplet $\{f'_i,g'_i,h'_i\}$ is taken from $W \times X \times Y$, *T* covers each element of *W*, *X* and *Y* exactly once, and for any $1\le i\le \frac{n}{3}$, $f'_i+g'_i+h'_i=b$ since $f_i+g_i+h_i=0$. $\square$

##### **Proposition 10**

*The* MZS3* problem is* $\frac{1}{3}$-*approximable*.

##### *Proof*

The approximation algorithm we provide here is a simple greedy algorithm we will call *A*, which repeats the following computation until $\mathcal {P}$ is empty: for each number *x* in $\mathcal {P}$, find two numbers *y* and *z* in $\mathcal {P}\setminus \{x\}$ such that $y+z=-x$. If such numbers exist, add triplet $\{x,y,z\}$ to the output set $\mathcal {P'}$ and remove *x*, *y* and *z* from $\mathcal {P}$; otherwise remove *x* from $\mathcal {P}$. We claim that *A* approximates MZS3 within a ratio of $\frac{1}{3}$. For this, consider an optimal solution, say Opt=$\{t_1,t_2\ldots t_m\}$ consisting of *m* independent triplets from $\mathcal {P}$ such that each sums to zero, and let us compare it to a solution Sol = $\{s_1,s_2\ldots s_k\}$ returned by *A*. First, note that any $t_i$, $1\le i\le m$ necessarily intersects with an $s_j$, $1\le j\le m$, otherwise $t_i$ would have been found by *A*, a contradiction. Moreover, any element of a triplet $t_i$ from Opt is present in at most one triplet from Sol. Now, it is easy to see that necessarily $m\le 3k$, since for any $1\le i\le m$, the three elements of a $t_i$ intersect with at least one and at most three different $s_j$s. Thus *A* achieves the sought approximation ratio of $\frac{1}{3}$. $\square$

##### **Theorem 11**

*The w problem is DCJ-* dist $\frac{4}{3}$-*approximable*.

##### *Proof*

Our approximation algorithm $A'$ considers the set $\mathcal {C}_u$ of unbalanced cycles and does the following: (a) find a maximum number of pairs of cycles whose imbalances sum to zero, and merge them by pairs, (b) among the remaining unbalanced cycles, find a maximum number of triplets of cycles whose imbalances sum to zero and merge them three by three, (c) merge the remaining unbalanced cycles into a unique (balanced) cycle. Once this is done, all cycles are balanced, and we know there exists an optimal way to obtain *n* balanced trivial cycles from this point (see Proposition 2). We note $n_2$ (resp. $n_3$) the number of cycles involved in the pairs (resp. triplets) of (a) [resp. (b)]. As previously discussed, $n_2$ can easily be computed, and $n_3$ is obtained by solving MZS3. We know that MZS3 is NP-complete (Proposition 9), and more importantly that MZS3 is $\frac{1}{3}$-approximable (Proposition 10) ; in other words, step (b) of algorithm $A'$ finds $n'_3\ge \frac{n_3}{3}$ (otherwise stated, $n'_3=\frac{n_3}{3}+x$ with $x\ge 0$) cycles that become balanced when merged by triplets. We will show in the rest of the proof that $A'$ approximates $wDCJ(g_1,g_2)$ within ratio $\frac{4}{3}$.

First let us estimate the number $m_{A'}$ of merges operated by $A'$. It can be seen that $m_{A'}=\frac{n_2}{2}+\frac{2n_3}{9}+\frac{2x}{3}+(n_u-n_2-(\frac{n_3}{3}+x)-1)$, and that after these merges have been done, we are left with $c'=n_b+\frac{n_2}{2}+\frac{n_3}{9}+\frac{x}{3}+1$ balanced cycles. Thus, by Proposition 2, the number of splits $s_{A'}$ that follow satisfies $s_{A'}=n-c'$, and the total number of wDCJ operated by $A'$, say $dcj_{A'}$, satisfies $dcj_{A'}=m_{A'}+s_{A'}=n-n_b+\frac{n_3}{9}+\frac{x}{3}+(n_u-n_2-\frac{n_3}{3}-x-2)$. In other words, since $x\ge 0$, we have that $dcj_{A'}\le n-n_b+n_u-n_2-\frac{2n_3}{9}$ [inequality (I1)]. Now let us observe an optimal sorting scenario of length $wDCJ(g_1, g_2)$, which, as we know by the results in “Main properties of sorting by wDCJ'', can be assumed to contain $m_{opt}$ merges followed by $s_{opt}$ splits. In any optimal scenario, the best case is when all of the $n_2$ cycles are merged by pairs, all of the $n_3$ cycles are merged by triplets, and the rest is merged four by four, which leads to $m_{opt}\ge \frac{n_2}{2}+\frac{2n_3}{3}+\frac{3(n_u-n_2-n_3)}{4}$. In that case, we obtain $c'_{opt}\le n_b + \frac{n_2}{2}+\frac{n_3}{3}+\frac{n_u-n_2-n_3}{4}$ balanced cycles, leading to $s_{opt}=n-c'_{opt}\ge n-n_b-\frac{n_2}{2}-\frac{n_3}{3}-\frac{n_u-n_2-n_3}{4}$ subsequent splits. Altogether, we conclude that $wDCJ(g_1,g_2)= m_{opt}+s_{opt}\ge n-n_b+\frac{n_3}{3}+\frac{n_u-n_2-n_3}{2}$, that is $wDCJ(g_1,g_2)\ge n-n_b+\frac{n_u}{2}-\frac{n_2}{2}-\frac{n_3}{6}$ [inequality (I2)].

Our goal is now to show that $dcj_{A'}\le \frac{4}{3}\cdot wDCJ(g_1, g_2)$. For this, it suffices to show that $4\cdot wDCJ(g_1, g_2)-3\cdot dcj_{A'}\ge 0$. Because of inequalities (I1) and (I2) above, $4\cdot wDCJ(g_1, g_2)-3\cdot dcj_{A'}\ge 0$ is satisfied whenever $S\ge 0$, where $S=4\cdot (n-n_b+\frac{n_u}{2}-\frac{n_2}{2}-\frac{n_3}{6})-3\cdot (n-n_b+n_u-n_2-\frac{2n_3}{9}).$ It can be easily seen that $S=n-n_b-n_u+n_2$. Note that we always have $n\ge n_b+n _u$ since *n* is the maximum possible number of cycles in $BG(g_1,g_2)$ ; besides, $n_2\ge 0$ by definition. Thus we conclude that $S\ge 0$, which in turn guarantees that our algorithm $A'$ approximates wDCJ-dist within the sought ratio of $\frac{4}{3}$. $\square$

### FPT issues concerning wDCJ-dist

Recall first that by Theorem 7, for any genomes $g_1$ and $g_2$, $wDCJ(g_1, g_2)=n-c+2m$, where *m* is the minimum number of cycle merges needed to obtain a set of balanced cycles from the unbalanced cycles of $BG(g_1, g_2)$. The NP-completeness of wDCJ-dist thus comes from the fact that computing *m* is hard, since *n* and *c* can be computed polynomially from $g_1$ and $g_2$. Computing *m* is actually closely related to the following problem:

#### Max-Zero-Sum-Partition (MZSP)

**Instance**: A multiset $\mathcal {S}=\{s_1,s_2\ldots s_n\}$ of numbers $s_i\in \mathbb {Z}^*$ s.t. $\sum _{i=1}^{n} s_i=0$.

**Output**: A maximum cardinality partition $\{S_1,S_2\ldots S_p\}$ of $\mathcal {S}$ such that $\sum _{s_j\in S_i} s_j=0$ for every $1\le i\le p$.

Indeed, let $\mathcal {C}_u=\{C_1,C_2\ldots C_{n_u}\}$ be the set of unbalanced cycles in $BG(g_1, g_2)$. If $\mathcal {S}$ represents the multiset of imbalances of cycles in $\mathcal {C}_u$, then the partition $\{S_1,S_2\ldots S_p\}$ of $\mathcal {S}$ returned by MZSP implies that for every $1\le i\le p$, $|S_i|-1$ cycles merges will be operated in order to end up with *p* balanced cycles. Thus, a total of $\sum _{i=1}^p (|S_i|-1)=n_u-p$ merges will be used. In other words, the minimum number of cycle merges *m* in the expression $wDCJ(g_1, g_2)=n-c+2m$ satisfies $m=n_u-p$, where *p* is the number of subsets of $\mathcal {S}$ returned by MZSP. Note that MZSP is clearly NP-hard, since otherwise we could compute $wDCJ(g_1, g_2)=n-c+2(n_u-p)$ in polynomial-time, a contradiction to Theorem 8.

A classical parameter to consider when studying FPT issues for a given minimization problem is the “size of the solution”. In our case, it is thus legitimate to ask whether wDCJ-dist is FPT in $wDCJ(g_1, g_2)$. However, it can be seen that $wDCJ(g_1, g_2)\ge m$ since $n-c$ is always positive, and that $m\ge \frac{n_u}{2}$ since all cycles in $\mathcal {C}_u$ are unbalanced and it takes at least two unbalanced cycles (thus at least one merge) to create a balanced one. Thus, proving that wDCJ-dist is FPT in $n_u$, as done in Theorem 12 below, comes as a stronger result.

##### **Theorem 12**

*The* wDCJ-dist *problem can be solved in* $O^*(3^{n_u})$ *, where* $n_u$ *is the number of unbalanced cycles in* $BG(g_1, g_2)$.

##### *Proof*

By Theorem 7 and the above discussion, it suffices to show that MZSP is FPT in $n=|\mathcal {S}|$, and more precisely can be solved in $O^*(3^n)$, to conclude. Indeed, if this is the case, then replacing $\mathcal {S}$ by the multiset of imbalances of cycles in $\mathcal {C}_u$ in MZSP (thus with $n=n_u$) allows us to compute *m*, and thus $wDCJ(g_1, g_2)$, in time $O^*(3^{n_u})$. Note first that MZSP is clearly FPT in *n*, just by brute-force generating all possible partitions of $\mathcal {S}$, testing whether it is a valid solution for MZSP, and keeping one of maximum cardinality among these. The fact that the complexity of the problem can be reduced to $O^*(3^n)$ is by adapting the Held-Karp Dynamic Programming algorithm [12, 13], which we briefly describe here. The main idea is to fill a dynamic programming table *D*(*T*, *U*), for any non-intersecting subsets *T* and *U* of *S*, where *D*(*T*, *U*) is defined as the maximum number of subsets summing to zero in a partition of $T\cup U$, with the additional constraint that all elements of *T* belong to the same subset. The number *p* that corresponds to a solution of MZSP is thus given by $D(\emptyset ,\mathcal {S})$. For any nonempty subset $X\subseteq \mathcal {S}$, we let $s(X)=\sum _{s_i\in X} s_i$. Table *D* is initialized as follows: $D(\emptyset ,\emptyset )=0$, $D(T,\emptyset )=-\infty$ for any $T\ne \emptyset$ such that $s(T)\ne 0$, and $D(T,U)=1+D(\emptyset ,U)$ for any $T\ne \emptyset$ such that $s(T)= 0$. Finally, the main rule for filling *D* is

$$\begin{aligned} D(T,U)=\max _{u\in U} D(T\cup \{u\},U\setminus \{u\}) \end{aligned}$$

It can be seen that computing any entry in table *D* is achievable in polynomial time, and that the number of entries is $3^n$. Indeed, any given element of *S* appears either in *T*, in *U*, or in $S\setminus (T\cup U)$: this can be seen as a partition of *S* into three subsets, and $3^n$ such partitions exist. Altogether, we have that *p* is computable in $O^*(3^n)$ – and this is also the case for the corresponding partition $\{S_1,S_2\ldots S_p\}$ of $\mathcal {S}$, that can be retrieved by a backward search in *D*. $\square$

### An integer linear programming for solving wDCJ-dist

The ILP we propose here actually consists in solving the MZSP problem. Once this is done, the number *p* of sets in the output partition is easily retrieved, as well as $wDCJ(g_1,g_2)$ since $wDCJ(g_1,g_2)=n-c+2(n_u-p)$, as discussed before Theorem 12. We also recall that $p\le \frac{n_u}{2}$, since it takes at least two unbalanced cycles to create a balanced one.

**Fig. 2:**
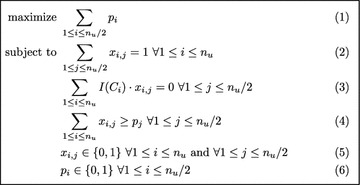
ILP description for the computation of parameter *p*

Our ILP formulation is given in Fig.  2 and described hereafter: we first define binary variables $x_{i,j}$, for $1\le i\le n_u$ and $1\le j\le \frac{n_u}{2}$, that will be set to 1 if the unbalanced cycle $C_i\in \mathcal {C}_u$ belongs to subset $\mathcal {C}_j$, and 0 otherwise. The binary variables $p_i$, $1 \le i \le \frac{n_u}{2}$, will simply indicate whether $\mathcal {C}_i$ is “used” in the solution, i.e $p_i=1$ if $\mathcal {C}_i\ne \emptyset$, and 0 otherwise. In our ILP formulation, (2) ensures that each unbalanced cycle is assigned to exactly one subset $\mathcal {C}_i$; (3) requires that the sum of the imbalances of the cycles from $\mathcal {C}_i$ is equal to zero. Finally, (4) ensures that a subset $\mathcal {C}_i$ is marked as unused if no unbalanced cycle has been assigned to it. Moreover, since the objective is to maximize the number of non-empty subsets, $p_i$ will necessarily be set to 1 whenever $\mathcal {C}_i\ne \emptyset$. Note that the size of the above ILP depends only on $n_u$, as it contains $\Theta (n_u^2)$ variables and $\Theta (n_u)$ constraints.

## A probabilistic model of evolution by wDCJ

In this section, we define a model of evolution by wDCJ, in order to derive theoretical and empirical bounds for the parameter $n_u$ on which both the FPT and ILP algorithms depend. The model is a Markov chain on all weighted genomes (that is, all weighted perfect matchings) on 2*n* vertices. Transitions are wDCJ, such that from one state, two distinct edges *ab* and *cd* are chosen uniformly at random, and replaced by either *ac* and *bd* or by *ad* and *cb* (with probability 0.5 each). Weights of the new edges are computed by drawing two numbers *x* and *y* uniformly at random in respectively [0, *w*(*ab*)] and [0, *w*(*cd*)], and assigning $x+y$ to one edge, and $w(ab)+w(cd)-x-y$ to the other (with probability 0.5 each).

### **Proposition 13**

*The equilibrium distribution of this Markov chain is such that a genome has a probability proportional to the product of the weights on its edges.*

### *Proof*

Define $\Pi$ as the probability distribution over the space of all genomes, such that for a genome *g*, $\Pi (g)$ is proportional to $\Pi _{e\in E(g)} w(e)$. Let $P(g_1, g_2)$ be the transition probability in the Markov chain between weighted genomes $g_1$ and $g_2$. We have that $P(g_1, g_2)=0$ unless $g_1$ and $g_2$ differ only by two edges, say *ab* and *cd* in $g_1$ and *ac* and *bd* in $g_2$. In that case, suppose wlog that $w(ab)<w(cd)$ and that $w(ac)=x+y$, where *x* and *y* are the numbers drawn by the model. We have two cases. If $w(ac) < w(ab)$, then $P(g_1, g_2)\sim w(ac)/(w(ab)w(cd))$ because there are exactly *w*(*ac*) combinations of *x* and *y* which can transform $g_1$ into $g_2$, over a total number of possibilities (*w*(*ab*)*w*(*cd*)); by the same reasoning, $P(g_2, g_1)\sim 1/w(cd)$, and if $w(ac) > w(ab)$, then $P(g_1, g_2)\sim 1/w(bd)$ and $P(g_2, g_1)\sim w(ab)/(w(ac)w(bd))$. In all cases, $\Pi (g_1)P(g_1,g_2)=\Pi (g_2)P(g_2, g_1)$, hence $\Pi$ is the equilibrium distribution of the Markov chain. $\square$

As a consequence, the weight distributions follow a symmetric Dirichlet law with parameter $\alpha =2$. It is possible to draw a genome at random in the equilibrium distribution by drawing a perfect matching uniformly at random and distributing its weights with a Gamma law of parameters 1 and 2.

We first prove a theoretical bound on the number of expected unbalanced cycles, and then show by simulations that this number probably stays far under this theoretical bound on evolutionary experiments.

### **Theorem 14**

*Given a weighted genome* $g_1$ *with nedges, if k random wDCJ are applied to* $g_1$ *to give a weighted genome* $g_2$ *, then the expected number of unbalanced cycles in* $BG(g_1, g_2)$ *satisfies* $\mathbb {E}(n_u)=O(k/\sqrt{n})$.

### *Proof*

In this proof, for simplicity, let us redefine the *size of a cycle* as half the number of its edges. Let $n_u^+$ (resp. $n_u^-$) be the number of unbalanced cycles of size greater than or equal to (resp. strictly less than) $\sqrt{n}$. We thus have $n_u=n_u^++n_u^-$. We will prove that (i) $n_u^+ \le k/\sqrt{n}$ and (ii) $\mathbb {E}(n_u^-) = O(k/\sqrt{n})$.

First, if the breakpoint graph contains *u* unbalanced cycles of size at least *s*, then the number *k* of wDCJ is at least *us*. Indeed, by Theorem 7 the wDCJ distance is at least $n-c+u$, and as $n\ge us + (c-u)$, we have $k \ge us + (c-u)-c+u = us$. As a consequence, $k\ge n_u^+\cdot \sqrt{n}$, and (i) is proved.

Second, any unbalanced cycle of size strictly less than *s* is the product of a cycle split. Given a cycle *C* of size $r>s$ with $r\not =2s$, there are *r* possible wDCJ which can split *C* and produce one cycle of size *s*. If $r=2s$, there are *r* / 2 possible splits which result in 2 cycles of size *s*. So there are *O*(*sr*) ways of splitting *C* and obtaining an unbalanced cycle of size less than *s*. If we sum over all cycles, this makes *O*(*sn*) ways because the sum of the sizes of all cycles is bounded by *n*. As there are $O(n^2)$ possible wDCJ in total, the probability to split a cycle of size *r* and obtain an unbalanced cycle of size less than *s* at a certain point of a scenario is *O*(*s*/*n*). If we sum over all the scenarios of *k* wDCJ, this makes an expected number of unbalanced cycles in *O*(*ks*/*n*), which implies (ii) since $s< \sqrt{n}$. $\square$

**Fig. 3:**
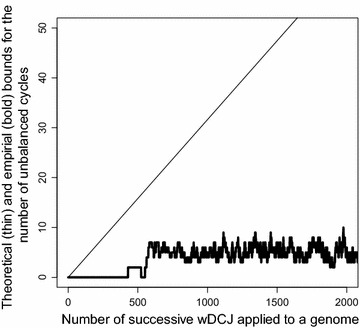
Number of unbalanced cycles (*y axis*), in a simulation on genomes with $n=1000$ edges where *k* wDCJ operations are applied successively (*k* is on the *x axis*). The number of unbalanced cycles is computed (i) according to the theoretical bound $k/\sqrt{n}$ (*in thin*), and (ii) directly from the simulated genomes (*in bold*)

We simulated a genome evolution with $n=1000$, and the weights on a genome drawn from the above discussed equilibrium distribution. Then we applied k=10,000 wDCJ, and we measured the value of $n_u$ on the way. As shown in Fig.  3 (up to $k=2000$ for readability), $n_u$ does not asymptotically grow with *k* (in the whole simulation a maximum of 13 was reached for *k* around 5500, while the mean does not grow up to k=10,000). This tends to show that the theoretical bound given in Theorem 14 is far from being reached in reality, and that parameter $n_u$ is very low is this model. We actually conjecture that the expected number $\mathbb {E}(n_u)=o(n)$ and in particular does not depend on *k*. Nevertheless, this shows that, in practice, both the FPT and ILP algorithms from the previous section should run in reasonable time on this type of instances. As an illustration, we ran the ILP algorithm described in Fig.  2 on a set of 10,000 instances generated as described above. For each of these instances, the execution time on a standard computer never exceeded 8 ms.

As a side remark, we note that the model presented here is different from the one used in Biller et al. [3], in which rearrangements are drawn with a probability proportional to the product of the weights of the involved edges. We checked that the behavior concerning $n_u$ was the same in both models ; however, we were unable to adapt proof of Theorem 14 to that case.

## Conclusion and perspectives

We made a few steps in the combinatorial study of rearrangement operations which depend on and affect intergene sizes. We leave open many problems and extensions based on this study. First, we would like to raise the two following algorithmic questions: is wDCJ-dist APX-hard? Can we improve the $O^*(3^{n_u})$ time complexity to solve wDCJ-dist? Second, the applicability of our model to biological data lacks additional flexibility, thus we suggest two (non exclusive) possible extensions: (a) give a weight to every wDCJ, e.g. a function of the weights of the involved edges; (b) instead of assuming that the total intergene size is conservative (which is not the case in biological data), consider a model in which intergene size may be altered by deletions, insertions and duplications—note that such a study is initiated in [9]. Third, generalizing the model to non co-tailed genomes (in our terminology, matchings that are not perfect) remains an open problem. It is clearly NP-complete, as it generalizes our model, but other algorithmic questions, such as approximability and fixed-parameter tractability, remain to be answered. Statistical problems are also numerous in this field. A first obvious question would be to improve the bound of Theorem 14, as it seems far from being tight when compared to simulations. Finally, we note that the present study compares two genomes with equal gene content, whereas realistic situations concern an arbitrary number of genomes with unequal gene content. This calls for extending the present work to more general models.

## Footnotes

1 The word *gene* is as usual in genome rearrangement studies taken in a liberal meaning, as any segment of DNA, computed from homologous genes or synteny blocks, which is not touched by a rearrangement in the considered history.
